# History of a Case of Varicella in a Lady, Producing Fatal Variola in Her Infant, and of the Case of Variola Being Followed by Varicella in One of the Same Family

**Published:** 1822-04

**Authors:** R. W. Bampfield

**Affiliations:** one of the Surgeons to the Royal Metropolitan Infirmary for Diseases of Children, &c.


### PATHOLOGY.

Art. II.—*History of a Case of Varicella in a Lady, producing fatal Variola in her Infant, and of the Case of Variola being followed by Varicella in one of the same Family.* By R. W. Bampfield, Esq. one of the Surgeons to the Royal Metropolitan Infirmary for Diseases of Children, &c.

As the following history of the contagion of varicella producing variola in the natural way, is strictly applicable to the interesting question which has, for some time, been agitated by the profession, respecting the identity of origin of chicken and small pox, I have deemed it incumbent on me to submit it to the public.

Mrs. Drury, 7, Henrietta-street, Covent Garden, had been vaccinated when young. On the 14th of December, 1821, she became affected with fever, head-ach, and nausea at the stomach; and, on the 16th, I was desired to see her, on account of those symptoms, and of an eruption of small red protuberances, differing in slight degrees in size and nearly circular, with a transparent vesicle in the centre, which had appeared since the preceding evening, and which were shown me, on the face, neck, breast, arms, and hands. I had no hesitation in pronouncing the eruption to be varicella. On the 17th, the eruption had spread all over her, and was painful in the palms of the hands. The febrile symptoms continued; the lymph of the vesicles on the extremities was quite transparent, but that of those on the face was not perfectly so; while all the vesicles, which were visible yesterday, had enlarged. On the 18th, the febrile symptoms had considerably abated; and the lymph in the vesicles first seen had become slightly straw-coloured. On the 19th, the vesicles on the face, generally, began to pucker and fade; and, in other parts, this stage of the vesicles was less generally observable. The febrile symptoms had almost wholly subsided. On the 20th, there was no fever; a small brown scab had begun to appear on some of the vesicles on the forehead and neck: and, on the 21st, all the vesicles had formed scabs, more or less perfectly, which, from the eighth to the eleventh day, fell off. From the vesicles on the palms of the hands producing pain, Mrs. D. was desired to puncture them with a needle, which had the effect of entirely evacuating the contained lymph, and of leaving them on a level with the surrounding integument, without their being again filled by a secreting process.

Mrs. Drury had been delivered of a male child on the 19th of October, which had not been vaccinated, and which she was suckling. This child had never been out of the house, nor exposed to variolous contagion in it. An eruption took place on Master Drury, (now two months old,) on the 25th of December, of numerous little red spots, on the face, neck, and breast: his eyes were red, his throat sore, and the febrile symptoms were rather severe. The eruption gradually spread to the extremities, and some eruptions appeared to come out successively, on parts of the body, for two or three days, where they had previously shown themselves.

On the 26th, the lips, the tongue, and the membranous lining of the mouth and fauces, were thickly studded with the eruption, and occasioned great difficulty in sucking and swallowing. The pocks were distinct, were surrounded by a circular margin of inflammation, and gradually filled and enlarged. The fever became moderate, although the irritation and restlessness were considerable and alarming. On the 29th, (the fifth day of the eruption,) it was evident the disease was not varicella, and the daily attendance of Dr. Granville was requested in consultation. The most superficial observer would now have pronounced the eruption to be small-pox: the face was swelled, the eyelids were nearly closed; the throat was very sore, and an occasional spasmodic affection of it sometimes threatened suffocation; the matter of the pustule was becoming opaque, and the pocks continued to extend, as well as the inflamed margin; the central depression was very evident; and it was discovered that the structure of the pock was cellular, and filled again on being punctured, by the mother having punctured many of the pocks on the palms of the hands and soles of the feet, to diminish irritation, without being enabled to empty them entirely, as she had her own. The irritation was great, and prevented all natural sleep; the child could no longer suck, and could only swallow its mother's milk, after being drawn from her breasts, with great difficulty. The fever was moderate. On the 30th, the eyes were quite closed, and the face and other parts more swelled. On the 31st, a few of the pustules on the face had broken, and the small-pox fetor was very distinct. On the 1st of January, 1822, the swelling on the face, &c. had evidently began to subside, and the pock to turn; the irritation had been allayed, so as to allow of sleep by small doses of anodyne medicines, prescribed by Dr. Granville; but the sore state of the lips, tongue, and fauces, produced by the variolous eruption, had prevented the infant from sucking; and, as the difficulty of swallowing had gradually increased, the child could not be sufficiently nourished, and died on the 2d of January.

On the same morning, (Jan. 2d,) I was called to Master Dickinson, a younger brother of Mrs. Drury, who had been living with her during this period of sickness, and had been formerly vaccinated. Fever had supervened the day before, and during the night the febrile symptoms were severe, and accompanied with head-ach and restlessness. An active purgative, followed by the use of neutral salts and antimon. tart., relieved the febrile symptoms. On the 3d, in the evening, three or four red spots were faintly seen on the face; which, on the 4th, exhibited a transparent vesicle in the centre, and the eruption had become more general over the body. This eruption was similar to Mrs. Drury's, and followed the same course. The vesicles, however, were not so numerous; neither did the febrile symptoms continue so distinct, or so long, as in his sister's case. From the fifth day, scabs successively formed on the vesicles, which began to fall off on the eighth day, (9th January,) when I withdrew my attendance. The central depression did not appear either in the pock of Mrs. Drury or Master Dickinson, neither did the throat become sore.

To whatever laws of evidence the above cases may be submitted, it appears to me that they must give some little additional weight to the opinions of Dr. M'Leod, Dr. Thomson, Mr. Cross, Mr. Black, &c. &c. in favour of the identity of origin of variola and varicella; and that they are in accordance with the experiments, by inoculation, of Dr. M'Leod, who has bestowed on the subject closer reasoning, and a stricter investigation, than any contemporary. (See *London Medical and Physical Journal*, for July and August 1820.)

*37, Bedford-street, Covent Garden;*
*March 9th, 1822.*

